# Effects of surface charge and boundary slip on time-periodic pressure-driven flow and electrokinetic energy conversion in a nanotube

**DOI:** 10.3762/bjnano.10.158

**Published:** 2019-08-06

**Authors:** Mandula Buren, Yongjun Jian, Yingchun Zhao, Long Chang, Quansheng Liu

**Affiliations:** 1 School of Mathematics and Statistics, Chifeng University, Chifeng, China; 2 School of Mathematical Science, Inner Mongolia University, Hohhot, China; 3 School of Mathematics and Statistics, Inner Mongolia University of Finance and Economics, Hohhot, China

**Keywords:** electroviscous effect, energy conversion, nanofluidics, streaming potential, surface charge-dependent slip

## Abstract

Time-periodic pressure-driven slip flow and electrokinetic energy conversion efficiency in a nanotube are studied analytically. The slip length depends on the surface charge density. Electric potential, velocity and streaming electric field are obtained analytically under the Debye–Hückel approximation. The electrokinetic energy conversion efficiency is computed using these results. The effects of surface charge-dependent slip and electroviscous effect on velocity and electrokinetic energy conversion efficiency are discussed. The main results show that the velocity amplitude and the electrokinetic energy conversion efficiency of the surface charge-dependent slip flow are reduced compared with those of the surface charge-independent slip flow.

## Introduction

Micro- and nanofluidic devices [1] have a wide range of applications in science and engineering, e.g., liquid pumping and energy conversion. In many of these devices, a pressure gradient is often used to manipulate the transport of electrolyte solutions in nanochannels, at least one characteristic dimension of which is below or of the order of 100 nm. The decrease of length scale of the channel leads to the emergence of new phenomena different from those in macroflow, such as the electrokinetic effect and boundary slip. When wall surfaces are brought into contact with an electrolyte solution, most of them acquire surface electric charge [2] due to ion adsorption and acid–base reactions [3]. The charged surface attracts counterions and repels co-ions in the nearby electrolyte solution, and hence an electric double layer (EDL) with net charge density forms in the nearby electrolyte solution. The flow of electrolyte solution actuated by the pressure field generates both a streaming current and a streaming potential. The streaming current in a nanochannel can offer a simple and effective way to convert the mechanical energy to electric energy [4]. The streaming potential induces an electric field called streaming electric field. Acting on the net mobile charge in EDL, the steaming electric field generates an electric force in the opposite direction of the flow. The flow rate is decreased under the action of the electric office. This effect is called electroviscous effect.

The electrokinetic energy conversion efficiency and the electroviscous effects of micro-and nanoscale flows have been widely studied under the no-slip assumption [4–10]. Among these works, Bhattacharyya et al. [7] discussed the electroviscous effect on a time-periodic pressure-driven flow through a circular microtube and have shown that the Onsager’s principle of reciprocity is applicable for this flow. The electrokinetic energy conversion efficiencies of time-periodic pressure-driven no-slip flows of a viscoelastic fluid between two parallel plates and in a circular tube were, respectively, by Bandopadhyay and Chakraborty [8] and Nguyen and co-workers [10]. The no-slip flow of a Maxwell fluid in a soft nanochannel and the electrokinetic energy conversion efficiency were studied by Jian and co-workers [9].

In nanoscale flow, the boundary slip effect becomes significant because the ratio between slip length and channel height becomes considerable large. For example, the velocity distribution for Stokes slip flow through a circular tube [11–12] is

[1] $$u=-\frac{a^{2}}{4\mu }\frac{\text{d}p}{\text{d}x}\left[1-\frac{r^{2}}{a^{2}}+\frac{2b_{0}}{a}\right],$$

where *a* is the radius of the circular tube and *b*_0_ is the Navier slip length. The last term 2*b*_0_/*a* becomes considerable large for nanoscale flow. Many researchers investigated the influences of the surface charge and the boundary slip on micro- and nanoscale flows [11–17]. Among these, Yang and Kwok [11–12] studied time-periodic pressure-driven flows in circular and parallel-plate microchannels with electrokinetic effect and boundary slip condition. Goswami and Chakraborty [17] investigated electrokinetic energy conversion through streaming effects in time-periodic pressure-driven nanochannel flows with boundary slip.

In the above mentioned references [11–17], the slip length is independent of the surface charge. However, recent theoretical and experimental results have shown that the surface charge affects the slip length. Joly et al. [18] used molecular dynamics simulations to find the relationship between slip length and surface charge density. The reason is that there exists an attracting electrostatic force between the charged solid surface and the liquid near the charged solid surface. They also described the coupling relationship between the surface charge and the slip length by a mathematical model in 2006. Experiments [19–21] showed that the slip length decreases as the absolute value of surface charge density increases. Many researchers [22–27] theoretically investigated the effects of the surface charge-dependent slip on the fluid flows and heat transfers in microtubes and parallel-plate microchannels. Recently, Buren et al. [28] studied the effect of surface charge-dependent slip and the electroviscous effect on time periodic pressure-driven flow and electrokinetic energy conversion in a parallel-plate nanochannel.

In the following sections, the influences of the surface charge-dependent slip on time-periodic pressure-driven flow and electrokinetic energy conversion in a circular nanotube are studied. To our knowledge, so far no author discussed this problem for the case of a time-periodic flow. Firstly, using the separation of variables method, the energy conversion efficiency and the analytical solutions to the governing equations are obtained. Secondly, the electroviscous effect and the effects of surface charge-dependent slip on the velocity and the energy conversion efficiency are discussed. Finally, we make our concluding remarks.

## Mathematical modeling

### Problem definition and governing equations

**Figure 1:**
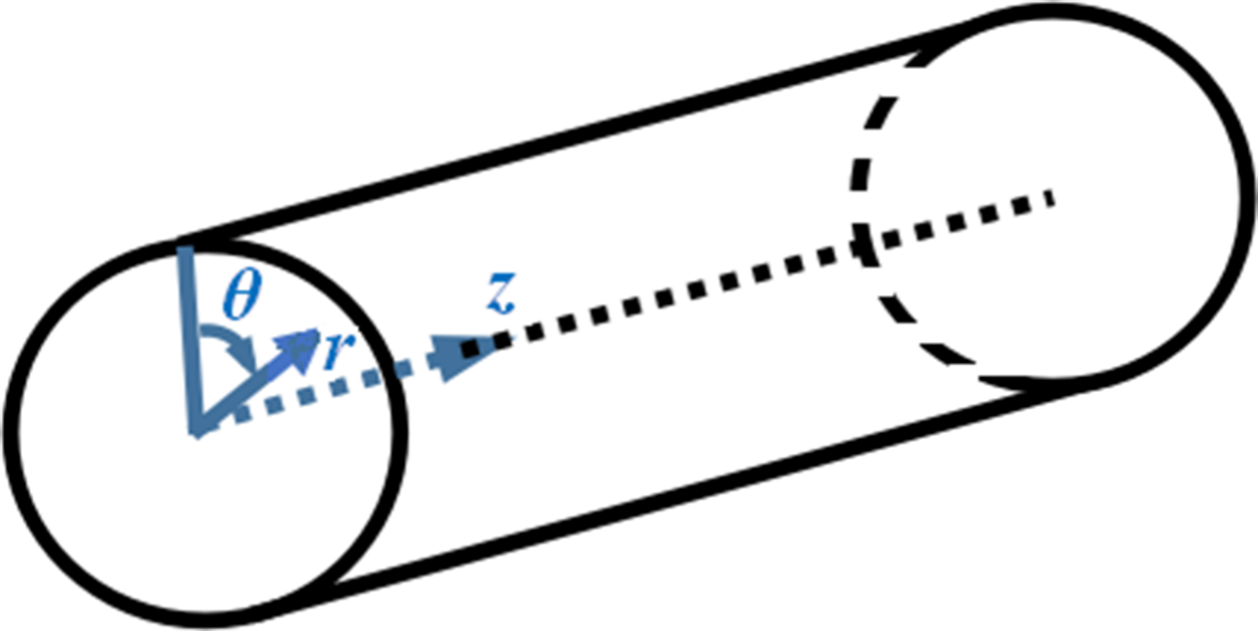
Schematic drawing of the circular nanotube.

We consider a time-periodic pressure-driven flow of a symmetric binary electrolyte in a circular nanotube with surface charge-dependent slip and with radius *a* using a cylindrical coordinate system (*r*, *θ*, *z*) where the *z*-axis is taken to coincide with the central axis of the nanotube, as shown in Figure 1*.* In the present study, the radius ranges from 37.5 to 100 nm, and the bulk ionic concentration varies from 1 to 100 nm. The corresponding Debye length varies from 0.96 to 9.63 nm [29]. In this case, the overlap of EDLs is mild, and hence the electric potential within the nanotube can be described by the Poisson–Boltzmann equation [30]. In addition, the continuum hydrodynamics for fluid flow is valid because the Debye length and the radius considered here are much larger than the mean free path of the liquid in the nanotube [31]. When the boundary surface of a nanochannel is molecularly smooth, the surface asperity barriers do not exist and, consequently, boundary slip can occur at the interface between the liquid and the boundary of the nanochannel [20]. Assume that the boundary slip *b* depends on the surface charge density *σ*_s_ [18,22,25]:

[2] $$b=\frac{b_{0}}{1+\left(1/\alpha \right){\left(\sigma_{\text{s}}d^{2}/e\right)}^{2}\left(l_{\text{B}}/d^{2}\right)b_{0}},$$

where *b*_0_ is the original slip length independent of surface charge density, α ≈ 1 is a numerical factor, *d* is the equilibrium distance of the Lennard–Jones potential, *e* is the elementary charge, *l*_B_ = *e*^2^/(4πε*k*_B_*T*), *k*_B_ is the Boltzmann constant, ε is the permittivity of the electrolyte solution and *T* is the absolute temperature [19,25].

The electric potential distribution φ in EDL satisfies the Poisson equation

[3] $$\frac{1}{r}\frac{\text{d}}{\text{d}r}\left(r\frac{\text{d}\varphi }{\text{d}r}\right)=-\frac{\rho_{e}}{\varepsilon },$$

where ρ*_e_* is the volumetric net charge density. When the number concentrations of the positive and negative ions in symmetric electrolyte in a nanochannel [17] obey the Boltzmann distribution, the volumetric net charge density ρ*_e_* can be obtained through the relationship

[4] $$\rho_{e}=-2ezn_{0}\sinh\left(ez\varphi /k_{\text{B}}T\right),$$

where *n*_0_ is the bulk ionic concentration and *z* is the valence of ions. Taking the Debye–Hückel approximation for low zeta potential, i.e., sinh(*ez*φ/(*k*_B_*T*)) ≈ *ez*φ/(*k*_B_*T*), and utilizing Equation 3 and Equation 4 we obtain the linearized Poisson–Boltzmann equation

[5] $$\frac{1}{r}\frac{\text{d}}{\text{d}r}\left(r\frac{\text{d}\varphi }{\text{d}r}\right)=\kappa^{2}\varphi .$$

The electric potential distribution φ satisfies

[6] $$\varphi {\text{|}}_{r=a}=\zeta \;,\;\;\varphi_{r=0}\text{ is finite}\text{,}$$

where ρ*_e_* = −εκ^2^*φ* is used, ζ is the zeta potential, and κ = [ε*k*_B_*T*/(2*z*^2^*e*^2^*n*_0_)]^−1/2^ is the Debye–Hückel parameter and represents the inverse of the characteristic EDL thickness.

A unidirectional flow along the *z*-direction is generated by a time-periodic pressure gradient cos(ω*t*)d*p*_0_/d*z* independent of position, where ω is the frequency, and *t* is the time. From the continuity equation, we find ∂*u*/∂*z* = 0 and so *u* depends on the variables *r* and *t*. Therefore, the momentum balance equations for the incompressible viscous Newtonian liquid becomes

[7] $$\rho \frac{\partial u}{\partial t}=-\frac{\partial p}{\partial z}+\mu \left(\frac{\partial^{\text{2}}u}{\partial r^{2}}+\frac{1}{r}\frac{\partial u}{\partial r}\right)+\rho_{e}E_{\text{s}},$$

where ρ is the mass density, μ is the dynamic viscosity, *E*_s_ is the streaming electric field and *p* = cos(ω*t*)*p*_0_ is the time-periodic pressure. The boundary conditions are

[8] $$u{|}_{r=a}=-b\frac{\partial u}{\partial r}{|}_{r=a},\;\;u{|}_{r=0}\text{ is finite}\text{,}$$

where the slip length *b* is given by Equation 2.

According to the charge conservation law, the total surface charge should be equal to the net charge in the fluid. Therefore

[9] $$2\pi aL\sigma_{\text{s}}=-\int_{0}^{a}\rho_{e}2\pi rL\text{d}r,$$

where *L* represents the length of the nanotube. From Equation 3, Equation 9 and the boundary condition in Equation 6, we obtain

[10] $$\sigma_{\text{s}}=\varepsilon \frac{\text{d}\varphi }{\text{d}r}{|}_{r=a}.$$

Substituting Equation 10 in Equation 2 yields

[11] $$b=\frac{b_{0}}{1+\left(1/\alpha \right){\left(\varepsilon \frac{\text{d}\varphi }{\text{d}r}{|}_{r=a}\frac{d^{2}}{e^{2}}\right)}^{2}\left(l_{\text{B}}/d^{2}\right)b_{0}}.$$

The streaming current through the channel is equal to

[12] $$I_{\text{s}}=2\pi \int_{0}^{a}ru\rho_{e}\text{d}r.$$

The streaming electric field *E*_s_ generates a reverse conduction current *I*_c_. Due to the electric neutrality of the fluid inside the nanotube, the net electric current over the cross section of the nanotube is zero, i.e.,

[13] $$I_{\text{s}}+I_{\text{c}}=0,\;\;I_{\text{c}}=\pi a^{2}\sigma E_{\text{s}},$$

where σ = 2*z*^2^*e*^2^*Dn*_0_/(*k*_B_*T*) is the electric conductivity and *D* is the diffusivity of ions in the electrolyte. From the equation *I_s_* + *I_c_*=0, the streaming electric field *E*_s_ can be obtained in the form:

[14] $$E_{\text{s}}=-\frac{2}{a^{2}\sigma }\int_{0}^{a}ru\rho_{e}\text{d}r.$$

### Dimensionless governing equations

The velocity and the streaming electric field of the time-periodic pressure-driven flow are both time-periodic functions and can be written as

[15] $$u=R\left[u_{0}\exp\left(-\text{i}\omega t\right)\right],\;\;E_{\text{s}}=R\left[E_{0}\exp\left(-\text{i}\omega t\right)\right],$$

where the *R*[·] denotes the real part of its complex argument and i = 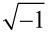. Let

[16] $$\begin{array}{c} \bar{r}=\frac{r}{a},\;\bar{\varphi }=\frac{\varphi }{\zeta },\;{\bar{u}}_{0}=\frac{u_{0}}{u_{\text{ref}}},\;u_{\text{ref}}=\frac{-a^{2}\text{d}p_{0}/\text{d}z}{4\mu },\\ {\bar{\rho }}_{e}=\frac{\rho_{e}}{-\varepsilon \kappa^{2}\zeta },\;\bar{b}=\frac{b}{a},\;{\bar{E}}_{0}=\frac{E_{0}}{E_{\text{ref}}},\;E_{\text{ref}}=\frac{-\varepsilon \zeta \text{d}p_{0}/\text{d}z}{4\sigma \mu }. \end{array}$$

Then, we use Equation 15 and Equation 16 to obtain the dimensionless governing equations and their boundary conditions:

[17] $$\frac{1}{\bar{r}}\frac{\text{d}}{\text{d}\bar{r}}\left(\bar{r}\frac{\text{d}\bar{\varphi }}{\text{d}\bar{r}}\right)={\left(\kappa a\right)}^{\text{2}}\bar{\varphi }\;,$$

[18] $$\bar{\varphi }{|}_{\bar{r}=1}=1,\;\;\bar{\varphi }{|}_{\bar{r}=0}\text{ is finite}\text{,}$$

[19] $$\frac{{\text{d}}^{2}{\bar{u}}_{0}}{\text{d}{\bar{r}}^{2}}+\frac{1}{\bar{r}}\frac{\text{d}{\bar{u}}_{0}}{\text{d}\bar{r}}+B^{2}{\bar{u}}_{0}=-4+\lambda {\bar{\rho }}_{e}{\bar{E}}_{0},$$

[20] $${\bar{u}}_{0}{|}_{\bar{r}=1}=-\bar{b}\frac{\text{d}{\bar{u}}_{0}}{\text{d}\bar{r}}{|}_{\bar{r}=1},\;\;{\bar{u}}_{0}{|}_{\bar{r}=0}\text{ is finite}\text{,}$$

where *λ* = εζ^2^/(µ*D*), *B*^2^ = iRe, and Re = ω*a*^2^ρ/μ is the nondimensional frequency. Using numerical methods, Erickson and Li [32] verified that the linearized Equation 17 and Equation 19 are also valid for large zeta potential values up to 100 mV.

### Analytical solutions of the governing equations

From the boundary value problem in Equation 17 and Equation 18, we obtain

[21] $$\bar{\varphi }=\frac{I_{\text{0}}\left(\kappa a\bar{r}\right)}{I_{\text{0}}\left(\kappa a\right)},$$

where *I*_0_ is the zeroth-order modified Bessel function of the first kind. The slip length is given by Equation 11 and Equation 21. From Equation 19 and Equation 20, the velocity distribution is obtained as

[22] $$\begin{array}{c} {\bar{u}}_{0}={\bar{u}}_{p_{0}}-\frac{\lambda {\bar{E}}_{\text{0}}}{I_{0}\left(\kappa a\right)\left[{\left(\kappa a\right)}^{2}+B^{2}\right]}\\ \cdot \left\{\frac{\left[I_{0}\left(\kappa a\right)+\bar{b}\kappa aI_{1}\left(\kappa a\right)\right]J_{0}\left(B\bar{r}\right)}{J_{0}\left(B\right)-\bar{b}BJ_{1}\left(B\right)}-I_{0}\left(\kappa a\bar{r}\right)\right\}, \end{array}$$

where *I*_1_ is the first-order modified Bessel function of the first kind, *J*_0_ and *J*_1_ are the zeroth-order and first-order Bessel functions of the first kind,

[23] $${\bar{u}}_{p_{0}}=\frac{4}{B^{2}}\left[\frac{J_{\text{0}}\left(B\bar{r}\right)}{J_{\text{0}}\left(B\right)-\bar{b}BJ_{\text{1}}\left(B\right)}-1\right]$$

is the complex velocity amplitude without considering the electroviscous effect.

From Equations 14–16 and Equations 21–23, we obtain

[24] E¯0=E¯p0/{1+2(κa)2λI02(κa)((κa)2+B2)[−12(I02(κa)−I12(κa))+(I0(κa)+b¯κaI1(κa)) (J0(B)κaI1(κa)+I0(κa)BJ(B)1)(J0(B)−b¯BJ1(B))((κa)2+B2)]},

where

[25] E¯p0=8(κa)2B2I0(κa){J0(B)κaI1(κa)+I0(κa)BJ(B)1[J0(B)−b¯BJ1(B)][(κa)2+B2]−I1(κa)κa}

is the complex electric field amplitude in which the electroviscous effect is not considered.

The dimensionless flow rate normalized by *a*^2^*u*_ref_ is given by

[26] $$\begin{array}{c} \bar{Q}=R\left[{\bar{Q}}_{0}\exp\left(-\text{i}\omega t\right)\right],\\ {\bar{Q}}_{0}=2\pi \int_{0}^{1}\bar{r}{\bar{u}}_{\text{0}}\text{d}\bar{r}\\ =\frac{8\pi }{B^{2}}\left[\frac{1}{J_{0}\left(B\right)-\bar{b}BJ_{1}\left(B\right)}\frac{J_{1}\left(B\right)}{B}-\frac{1}{2}\right]\\ -\frac{\lambda {\bar{E}}_{0}}{I_{0}\left(\kappa a\right)\left[{\left(\kappa a\right)}^{2}+B^{2}\right]}\\ \cdot \left[\frac{I_{0}\left(\kappa a\right)+\bar{b}\kappa aI_{1}\left(\kappa a\right)}{J_{0}\left(B\right)-\bar{b}BJ_{1}\left(B\right)}\frac{J_{1}\left(B\right)}{B}-\frac{I_{1}\left(\kappa a\right)}{\kappa a}\right]. \end{array}$$

### Energy conversion efficiency

The mechanical energy of the pure pressure-driven flow is converted into electric energy [6] when the streaming current (*I*_s_) and the streaming electric field (*E*_s_) are generated. The energy conversion efficiency *η* [10]can be expressed as:

[27] $$\eta =\frac{1}{4}\frac{\langle I_{\text{s}}E_{\text{s}}\rangle }{\langle \frac{\partial p}{\partial x}Q_{\text{p}}\rangle },$$

where ⟨·⟩ represents the time average over one cycle [0, 2π/ω], and

[28] $$Q_{\text{p}}=\int_{0}^{a}R\left[u_{\text{ref}}{\bar{u}}_{p_{0}}\text{exp}\left(-\text{i}\omega t\right)\right]\text{2}\pi r\text{d}r$$

is the flow rate of the pure pressure-driven flow without considering the effect of the surface charge on the slip length.

Using Equation 13, Equation 15, Equation 28 and the non-dimensional definitions in Equation 16, we can obtain the following expression for η from Equation 27:

[29] $$\eta =\frac{\pi }{16}\frac{\lambda }{{\left(\kappa h\right)}^{2}}\frac{{\left|{\bar{E}}_{0}\right|}^{2}}{\left|{\bar{Q}}_{p_{0}}\right|\cos\left(\arg({\bar{Q}}_{p_{0}})\right)},$$

where

[30] $${\bar{Q}}_{p_{0}}=\frac{8\pi }{B^{2}}\left[\frac{1}{J_{0}\left(B\right)-\bar{b}BJ_{1}\left(B\right)}\frac{J_{1}\left(B\right)}{B}-\frac{1}{2}\right],$$

and 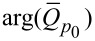 is the principal argument of 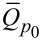.

## Results and Discussion

**Figure 2:**
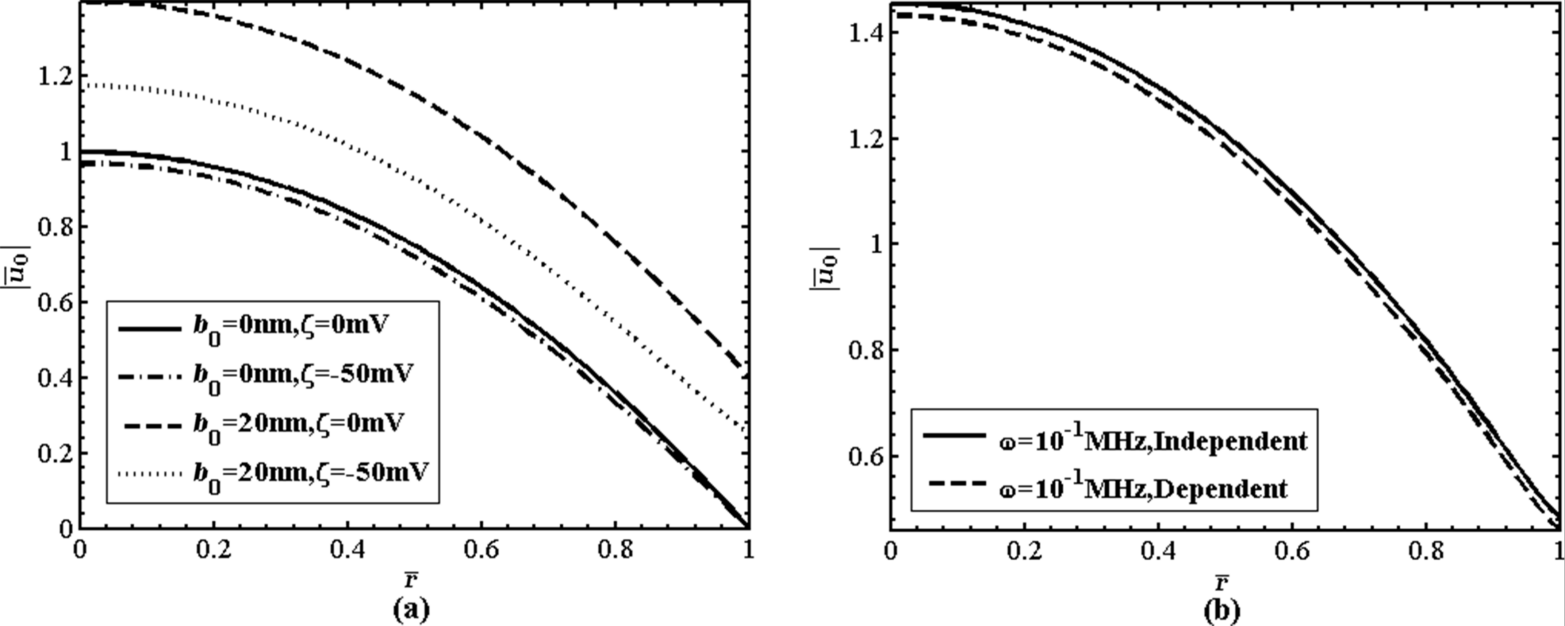
(a) The coupling influences of the boundary slip and the zeta potential on the velocity amplitude (ω = 10^−1^ MHz, *n*_0_ = 1 mM, *a* = 100 nm); (b) Comparison between the velocity amplitude of the surface charge-dependent slip flow and that of the surface charge-independent slip flow (ζ = −50 mV, *b*_0_ *=* 20 nm, *n*_0_ = 100 mM, *a* = 37.5 nm).

This section discusses the effects of the surface charge-dependent boundary slip on the time-periodic pressure-driven flow and electrokinetic energy conversion efficiency in the nanotube using the analytical results obtained above. In this problem, we assume that [2,8,19,25]: α = 1, *d* = 0.4 × 10^−9^ m, µ = 1.01 × 10^−3^ Pa·s, ρ = 1 × 10^3^ kg·m^−3^, *e* = 1.6 × 10^−19^ C, *z* = 1, *D* = 1.612 × 10^−9^ m^2^·s^−1^, *T* = 298 K, ε = 7 × 10^−10^ F·m^−1^, and *k*_B_ = 1.38 × 10^−23^ J·K^−1^. Figure 2 shows the variation of the velocity amplitudes with the radial coordinate *r̄* at different values of zeta potential, slip length and frequency. Figure 2a shows that, for the no-slip and slip flows, the amplitudes of the velocity are both reduced because of the electroviscous effect. The reduction of velocity amplitude of slip flow is larger than that of no-slip flow. The reason is that the slip length is decreased by the surface charge effect and, consequently, the velocity of slip flow is further reduced. It is apparent in Figure 2b that due to the surface charge effect, the velocity amplitude of the surface charge-dependent slip flow is less than that of the surface charge-independent slip flow.

**Figure 3:**
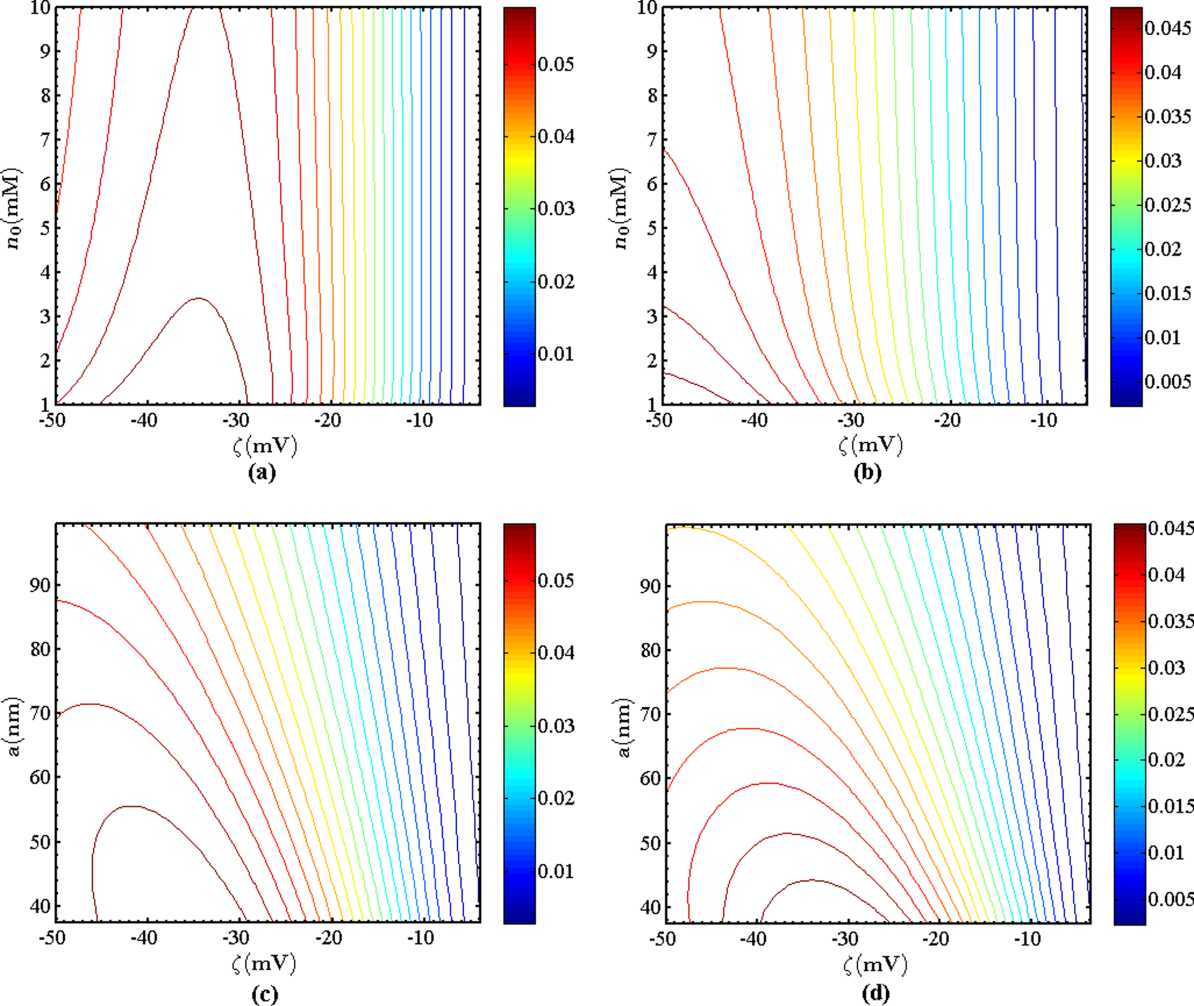
(a, b) Contours of the electrokinetic energy conversion efficiency (ω = 10^−1^ MHz, *b*_0_ = 40 nm) for different radii: (a) *a* = 37.5 nm and (b) *a* = 100 nm; (c, d) contours of the electrokinetic energy conversion efficiency (ω = 10^−1^ MHz, *b*_0_ = 40 nm) for different ionic concentrations: (c) *n*_0_ = 1 mM and (d) *n*_0_ = 100 mM.

Figure 3 shows the contours of the electrokinetic energy conversion efficiency in the circular nanotube with surface charge-dependent slip. In the low ionic concentration regime, the electrokinetic energy conversion efficiency varies quickly with the ionic concentration and is highest. Note that for the low ionic concentration, κ*a* is small. The variation of the efficiency with *a* is not monotonous, but the efficiency is large when κ*a* is small. This is because as κ*a* decreases, the ions in the EDL extend into the middle of the nanotube, where the velocity of the flow is highest. Figure 3 shows that the electrokinetic energy conversion efficiency is large in the regime where the zeta potential is large.

**Figure 4:**
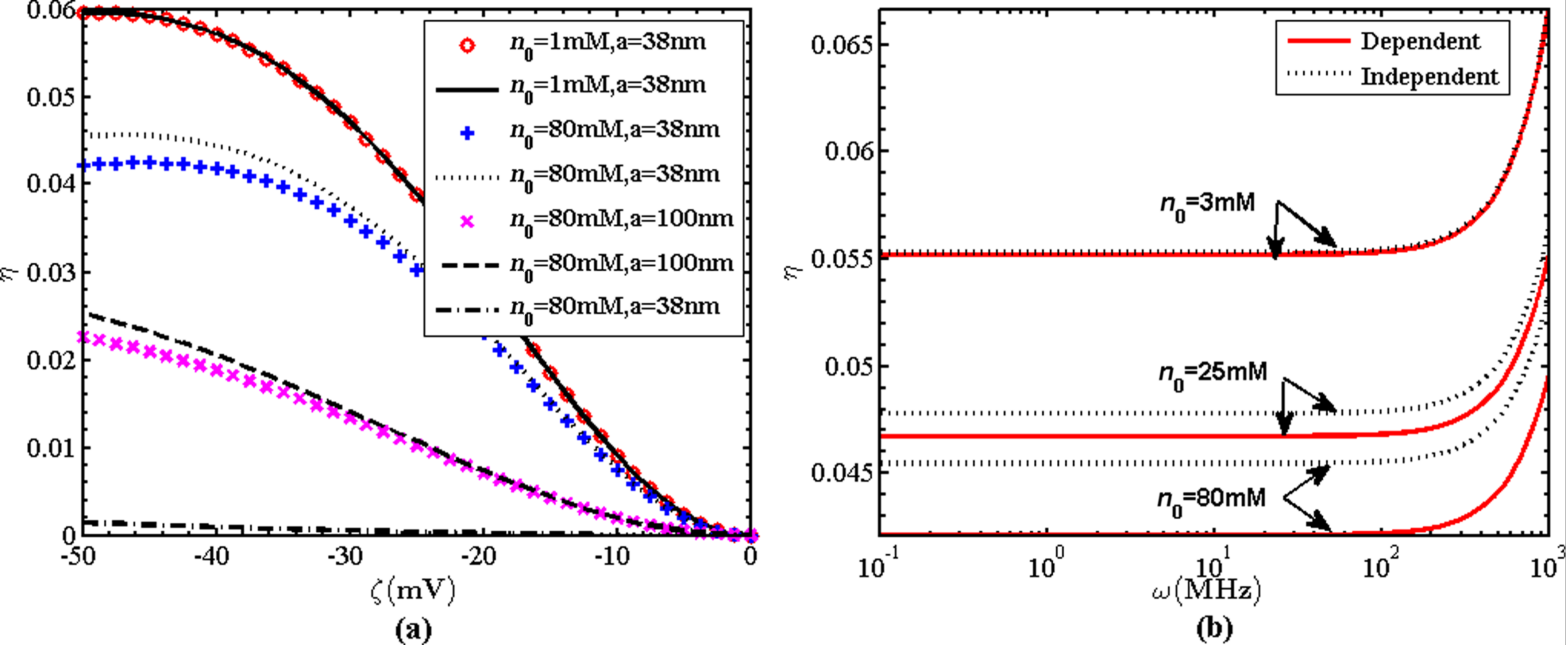
(a) Variation of η as function of ζ at different values of *a* and *n*_0_ (ω = 10^−1^ MHz, *b*_0_ = 20 nm) under the condition of surface charge-dependent slip (circles, plus signs and diagonal crosses), surface charge-independent slip (solid, dashed and dotted lines) and no-slip (dashdot line); (b) variation of η as function of ω for the charge-dependent and charge-independent cases (*a* = 38 nm, *b*_0_ = 20 nm).

Figure 4a shows the comparison of the electrokinetic energy conversion efficiency values under the condition of surface charge-independent slip (solid, dashed and dotted lines), surface charge-dependent slip (circles, plus signs and diagonal crosses) and no-slip (dashdot line). The boundary slip enhances the electrokinetic energy conversion efficiency. This is because the boundary slip increases the fluid velocity and the transportation of ions in the EDL, increasing the streaming electric field. Hence, the ratio of electric energy to mechanical energy, namely, the electrokinetic energy conversion efficiency is enhanced by the boundary slip. In fact, the increases in velocity and streaming electric fields increase both electric energy and mechanical energy, but the former is larger than the latter. Figure 4a,b shows that the electrokinetic energy conversion efficiency is reduced when the slip length depends on the surface charge. The reduction in the electrokinetic energy conversion efficiency is large when the ionic concentration and the zeta potential are large. This is caused by the slip length reduction due to the surface charge effect, as shown in Equation 11. In addition, Figure 4b shows that the electrokinetic energy conversion efficiency increases with the frequency when the frequency is larger than 10^2^ MHz; elsewhere the electrokinetic energy conversion efficiency is almost constant.

## Conclusion

In this paper, we apply the separation of variables method to study time-periodic pressure-driven flow and electrokinetic energy conversion efficiency in a circular nanotube with surface charge-dependent slip. The expressions for the electric potential, velocity distribution, streaming electric field, flow rate and electrokinetic energy conversion efficiency are obtained analytically. From the above theoretical results the following conclusions are drawn: Compared with the velocity amplitude of the pure pressure-driven flow, the velocity amplitudes of the no-slip and surface charge-dependent slip flows are both reduced. This is caused by the electroviscous effect. The decrement in the slip length due to surface charge effect causes the velocity amplitude of the surface charge-dependent slip flow to be smaller than that of the surface charge-independent slip flow. The effect of surface charge on the time-periodic flow increases with the magnitude of the zeta potential and with the ionic concentration. The electrokinetic energy conversion efficiency is large when the ratio between the channel radius and EDL thickness is small. Higher frequency leads to higher electrokinetic energy conversion efficiency. The boundary slip increases the electrokinetic energy conversion efficiency. The electrokinetic energy conversion efficiency is reduced when the slip length is dependent on the surface charge. The reduction in electrokinetic energy conversion efficiency due to surface charge effect is large for the cases of large ionic concentration and large zeta potential.
